# Evaluation and Control of Variability in RAP Properties Through Refined Fractionation Processing Methods

**DOI:** 10.3390/ma18214944

**Published:** 2025-10-29

**Authors:** Yan Zhang, Jiyang Li, Yiren Sun

**Affiliations:** 1 City Institute, Dalian University of Technology, Dalian 116600, China; 2 School of Infrastructure Engineering, Dalian University of Technology, Dalian 116024, China

**Keywords:** reclaimed asphalt pavement, variability, fractionation process, gradation, asphalt concrete

## Abstract

Variability in reclaimed asphalt pavement (RAP) properties, such as aggregate gradation, asphalt content, and moisture content, poses a significant challenge to producing consistent and reliable recycled asphalt mixtures. This study systematically evaluated processing techniques for mitigating variability through a comparative analysis of four fractionation strategies, i.e., unfractionated, two-fraction, four-fraction, and six-fraction processing. Corresponding to the four approaches, four distinct reference RAP mixtures were fabricated by proportionally recombining the obtained RAP fractions towards a target gradation. The gray relational analysis (GRA) was employed to quantify geometric similarity between the gradation curve of reclaimed aggregates from each fraction and the target gradation curve, thereby facilitating efficient determination of blending proportions without resorting to complex optimization algorithms. Statistical variability indicators, including range, standard deviation, and coefficient of variation (COV), were used to assess the effectiveness of each fractionation and recombining method. The results demonstrated that refined fractionation processing significantly reduced variability, particularly in gradation properties. Compared with the COV values from the commonly used two-fraction processing, those from the refined four-fraction and six-fraction processing methods decreased by up to 51.5% and 73.5%, respectively. While increasing the number of fractions generally enhanced homogeneity, the four-fraction approach emerged as the most technically reliable and economically viable strategy, achieving a desirable balance between processing effort and variability control. Furthermore, the GRA proved to be a practical and efficient tool for blend proportioning, reducing reliance on complex numerical methods. These findings reveal the importance of refined fractionated RAP processing in enabling the production of high-RAP recycled mixtures with improved uniformity and performance.

## 1. Introduction

The utilization of reclaimed asphalt pavement (RAP) in new asphalt mixtures is growing increasingly widespread within the pavement industry [1,2,3]. The RAP material, obtained by milling or removing deteriorated pavements, consists of valuable crushed aggregates and hardened aged binder. Its reuse supports natural resource conservation and cost reduction, thus offering considerable economic and environmental advantages. In recent years, the pursuit of sustainable pavement infrastructure has substantially increased the motivation to use high percentages of RAP in recycled mixtures [4,5,6]; however, one major concern is variability in RAP properties like aggregate gradation, asphalt content, and moisture content, which poses a severe challenge to the quality control of high-RAP recycled mixtures [7,8,9]. Montanez et al. [10] reported that variability in RAP materials prominently affects the moisture susceptibility of recycled mixes. Austerman et al. [11] further demonstrated that even low RAP contents (e.g., <15%) can lead to significant performance deviations if the variability of RAP is not properly controlled. As a consequence, many highway agencies restrict the RAP content in surface layers to levels below 25% to lower these potential risks [12,13].

Reducing the variability in RAP is critical to ensuring the performance of recycled mixtures, especially those with high RAP contents, since it not only complicates mix design but also adversely influences the homogeneity of mixtures [11,14,15]. There exist numerous factors that lead to RAP variability [16]. Typically, RAP materials are sourced from extensive pavement sections within a region, where variations in original material specifications, construction dates, traffic history, and environmental exposure create inherent heterogeneity. Furthermore, additional variability can be introduced during the milling and collection process. Finally, stockpiling, transportation, and handling operations can induce segregation and further alter material properties, compounding the overall variability [17,18]. From a mesoscopic viewpoint, both the gradation of old aggregates and the content and properties of aged binder in RAP vary greatly, and the RAP particle agglomeration further intensify the uncertainty of particle gradations and inconsistency of activated binder contents in recycling process [19,20,21,22,23].

To address the variability issue, researchers have explored various management and processing approaches for RAP materials [24,25]. Currently, appropriate management strategies primarily involve milling in layers to segregate diverse mix types, separating RAP into multiple stockpiles for different sources, and maintaining homogeneity by mixing RAP in stockpiles [18]. Although these practices are valuable, they mostly fail to capture the quantitative variation details on RAP consistency throughout homogenization operations. By contrast, processing methods, primarily through crushing (using impact crushers, jaw crushers, etc.) and fractionation (with various sieve sequences), are more straightforward strategies for reducing variability. These processing procedures are able to effectively break down agglomerations and control maximum particle size, thereby transforming variable RAP into a consistent recycled material. In addition, emerging techniques such as refined decomposition of aged binder and old aggregates in RAP offer future alternatives [26,27].

Among the aforementioned approaches, the fractionation processing for RAP materials, which divides the collected RAP into two or more stockpiles by particle size, appears to be the most commonly used one [28,29]. Research efforts have been made to evaluate and control the variability of RAP by various fractionation processing methods. Shen et al. [30] employed the 4.75 mm sieve as a critical sieve for two-fraction processing and the 4.75 mm and 2.36 mm sieves for the three-fraction processing. They reported that in terms of the Superpave volumetric requirements, incorporating an additional 2.36 mm sieve effectively improved the possible content of RAP by about 30%. To alleviate the negative effect of excessive fine aggregates in RAP, Shannon et al. [28] designed two dual-fraction (coarse and fine fractions) processing methods, and found that owing to the improved aggregate structure, the optimum asphalt content was lowered when the fractionation processing was involved. Katla et al. [4] analyzed the influence of different fractionating practices, including unfractionation, two-level fractionation, and three-level fractionation, and showed that three-level fractionation processing resulted in much lower variability in the volumetric properties and performance of recycled mixtures than those from the unfractionation and two-level fractionation methods.

Although the benefits of fractionation processing in reducing the RAP variability has been widely accepted, existing studies and practices mostly concentrated on the two-fraction method and detailed and quantitative investigations into the efficacy of refined fractionation processing operations remain very limited [4,28,29]. Thus, there is an urgent need to develop new methods that can appropriately evaluate and control RAP variability. To this end, this study presented an approach to effectively evaluate the effect of refined fractionation processing operations on the variability in properties of RAP materials as well as a recombining method by means of gray relational analysis (GRA) to accurately determine the blending proportions of all RAP fractions so that desired properties can be well achieved.

## 2. Materials

The RAP material utilized in this study was collected from the binder layer (middle surface layer) of an expressway located in Dalian, China. The material was obtained via cold milling of the asphalt pavement, which was designed as an AC-16 mixture with a nominal maximum aggregate size (NMAS) of 16 mm. The original binder was an unmodified asphalt with a penetration grade of 80/100, and the original coarse aggregates were basalt. The moisture content of the RAP material was measured as 1.27% in accordance with the test methods of aggregates for highway engineering of China [31]. In this study, the recycled asphalt mixture was considered as AC-16 with a NMAS of 16 mm.

**Figure 1:**
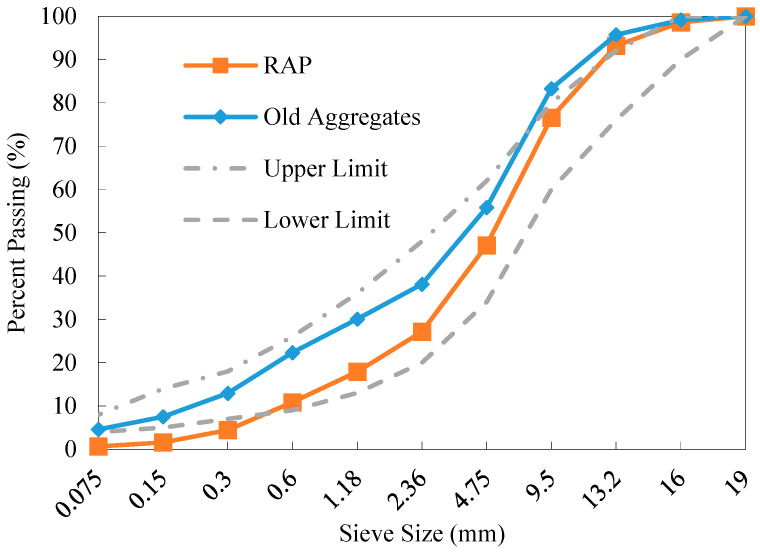
Gradations of the RAP material and extracted old aggregates.

The aged asphalt binder was extracted from the RAP material using trichloroethylene solvent, and recovered from the solution via a rotary evaporator [32]. In this manner, the asphalt content in the RAP was determined as 4.02% and the old RAP aggregates were obtained. The basic properties of the aged asphalt and old coarse aggregates in the RAP material are presented in Table 1. As observed, all the measurements of the properties satisfy the requirements for recycling [33]. Sieve analysis was conducted on both the RAP material and the reclaimed RAP aggregates. The resulting gradations are shown in Figure 1. As can be seen, the size distribution of the coarse aggregates in the RAP experienced significant variation due to long-term traffic loading and the mechanical action during milling.

## 3. Methodology

### 3.1. Fractionation Processing Methods

Fractionation is one of the most commonly used processing approaches for mitigating the variability in RAP properties. In current engineering practice, RAP is generally processed into two fractions, i.e., the coarse and fine ones; however, there would be still considerable heterogeneity in recycled mixtures, especially for the utilization of high percentages of RAP. For this reason, refined fractionation methods were adopted to achieve better control of the RAP variability.

In the present study, four scenarios were taken into account to investigate the effect of various fractionation processing methods. In Scenario 1, the RAP was unfractionated; that is, the particle size of the RAP ranged from 0 to 19 mm. In Scenario 2, the RAP material was separated into two stockpiles by the sieve of 4.75 mm as the common practice, resulting in two fractions, 0~4.75 mm and 4.75~19 mm. The sieve of 4.75 mm was chosen as a critical sieve mainly due to a common practice for aggregate fractionation processing in China. In Scenario 3, the fractionation process for the RAP was refined by four stockpiles, namely, 0~4.75 mm, 4.75~9.5 mm, 9.5~13.2 mm, and 13.2~19 mm. In Scenario 4, six detailed fractions were considered, i.e., 0~2.36 mm, 2.36~4.75 mm, 4.75~9.5 mm, 9.5~13.2 mm, 13.2~16 mm, and 16~19 mm. After fractionation processing, the fractionated RAP materials were recombined towards a target gradation. In this study, the average gradation (median) between the upper and lower limits for AC-16 was taken as the target gradation for the blended aggregates of the recycled mixture.

### 3.2. Proportioning RAP Fractions into Reference Materials

Corresponding to the four fractionation processing methods mentioned above, four different reference RAP materials, denoted as Reference-1, Reference-2, Reference-3, and Reference-4, were obtained by proportioning the RAP factions in terms of the target gradation, as listed in Table 2. The desired gradation can be attained by combining the old aggregates recovered from the reference RAP materials and the virgin aggregates that have been divided into sieve sizes. In this way, the variability in RAP can be well controlled, and the effects of different fractionation methods can be revealed in detail.

To obtain the optimal proportions of the RAP fractions, the GRA [34,35] was employed in this study. The GRA, which was proposed by Professor Julong Deng, offers notable advantages, including flexibility with sample size, minimal distribution requirements, low computational cost, and consistent integration of quantitative and qualitative results. The underlying principle of the GRA is that the strength of the relationship between data sequences is governed by the similarity of their geometric curves. Greater geometric similarity corresponds to a stronger relational grade. As such, the RAP fraction with greater similarity in the gradation curve of old aggregates to the target gradation curve can be considered to account for a larger proportion in the reference RAP material. Based on this theory, the proportions of all fractions of old aggregates can be rationally and readily determined. Detailed information about GRA can be found elsewhere [34].

Since Reference-1 was an unfractionated material, it was taken as a control material and the GRA was applied only to References-2 through 4. Before performing the GRA, the old aggregates in all the RAP fractions from different fractionation processing methods were recovered by the ignition method [36]. The specific computational steps are described as follows:

(1) Let *Y*(*i*) be the percent passing at each sieve size for the target gradation, and *X_j_*(*i*) be the percent passing of the *j*-th fractionated RAP material at the corresponding sieve size, where *i* denotes the sieve number (*i* = 1, 2, …, *n*) and *j* represents the number of the RAP fractions (*j* = 1, 2, …, *k*). For example, the gradations of the old aggregates from the six-fraction processing method are shown in Table 3. Since the data sequences shared the same dimensionality, the conventional normalization process was omitted in this study.

(2) Calculate the difference sequences *d_j_*(*i*) between *Y*(*i*) and *X_j_*(*i*), as follows:

(1) $$d_{j}(i)=|Y(i)-X_{j}(i)|$$

For instance, the difference sequences for the Reference-4 RAP blend is presented in Table 4.

(3) Determine the maximum value (*d*_max_) and minimum value (*d*_min_) of the difference sequences:

(2) $$d_{\max}=\underset{j}{\max}\;\underset{i}{\max}\;d_{j}\left(i\right)$$

(3) $$d_{\min}=\underset{j}{\min}\;\underset{i}{\min}\;d_{j}\left(i\right)$$

The calculated values are shown in Table 5.

(4) Calculate the gray relational coefficient *λ_j_*(*i*), gray relational degree *λ_j_*, and proportion of each fraction of the old aggregates *ρ_j_* using the following equation:

(4) $$\lambda_{j}(i)=\frac{d_{\min}+\xi d_{\max}}{d_{j}(i)+\xi d_{\max}}$$

(5) $$\lambda_{j}=\frac{\sum_{i=1}^{n}\lambda_{j}(i)}{n}$$

(6) $$\rho_{j}=\frac{\lambda_{j}}{\sum_{j=1}^{k}\lambda_{j}}$$

where *ξ*∈(0,1) is the distinguishing coefficient, and in this study *ξ* is assumed to be 0.3. Table 6 displays the calculation results for the Reference-4 RAP material. It can be seen that the gradation of Fraction 2 has the greatest relational degree with the target gradation, followed by those of Fractions 3 and 5.

(5) Let *Z*(*i*) and *T*(*i*) be the percent passing of old aggregates of a reference RAP blend and virgin aggregates at the *i*-th sieve. Calculate the gradations of the reference RAP blends and the corresponding ones of the virgin aggregates at a RAP content *M* using the following equations:

(7) $$Z(i)=\sum_{j=1}^{k}[X_{j}(i)\times \rho_{j}]$$

(8) $$T(i)=\frac{Y(i)-M\times Z(i)}{1-M}$$

**Figure 2:**
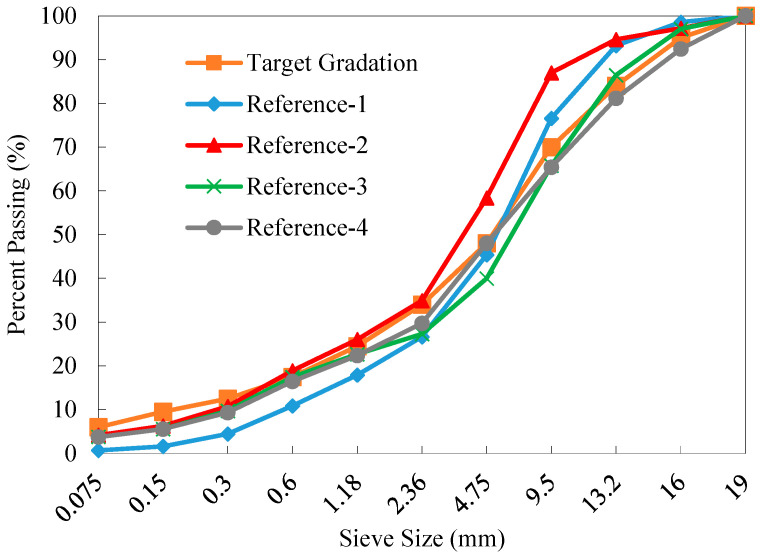
Calculated gradations of old aggregates of the four reference RAP materials.

Figure 2 shows the calculated gradations of reclaimed aggregates of the four reference RAP materials. It can be observed that as the number of fractions increased, the gradation curve of old aggregate blends approached closer to the target gradation curve. The old aggregate gradation curves of References-3 and 4 were nearly identical, which indicates that from the perspective of aggregate blending, the processing methods that fractionate the RAP material into four and six fractions yielded comparable effects. Therefore, dividing RAP material into four fractions is more economically viable based solely on the desired gradation. It can be found that the GRA offers a simple and efficient approach for directly determining the proportion of each RAP fraction, avoiding the dependence on the nonlinear optimization algorithm for minimizing a target error function.

### 3.3. Determining Moisture Contents of RAP Materials

RAP materials contain a certain amount of moisture. The presence of moisture not only prolongs the heating time of RAP, leading to further aging of the old asphalt, but also increases energy consumption during construction, thereby raising production costs. To ensure effective heating of RAP materials and improve production efficiency, the moisture content of RAP, *w*, is required to be less than 3% according to the technical specifications for highway asphalt pavement recycling of China [33], in which *w* is defined by the following:

(9) $$w=\frac{m_{w}-m_{d}}{m_{d}}\times 100$$

where *m*_w_ is the original mass of RAP (around 2000 g), and *m*_d_ is the constant mass of RAP after being dried in an oven at 105 °C.

### 3.4. Determining Asphalt Contents of RAP Materials

The asphalt contents of the RAP materials were determined using the ignition method [36]. A sample of RAP (about 2000 g) is weighed and placed in a furnace at 105 °C until it is dried to a constant mass. The mass of the dried RAP is recorded as *m*_1_. The dried sample is then transferred to a preheated ignition furnace, where it is subjected to high temperatures between 482 °C and 538 °C until a constant mass *m*_2_ is achieved. The mass loss is recorded as *m*_1_ − *m*_2_. The asphalt content of the RAP, *Pb*, is finally calculated by the following:

(10) $$Pb=\frac{m_{1}-m_{2}}{m_{1}}\times 100$$

### 3.5. Evaluating RAP Variability

The coefficient of variation (COV) was adopted as the indicator for evaluating the RAP variability. The value of *COV* is jointly influenced by both the standard deviation *S* and the mean $\bar{x}$. Therefore, when using the *COV* to assess the uniformity of a dataset, both the mean $\bar{x}$ and the standard deviation *S* should be reported together. The *COV* is expressed by the following:

(11) $$COV=\frac{S}{\bar{x}}\times 100$$

(12) $$\bar{x}=\frac{\sum_{\alpha =1}^{N}x_{\alpha }}{N}$$

(13) $$S=\sqrt{\frac{\sum_{\alpha =1}^{N}{\left(x_{\alpha }-\bar{x}\right)}^{2}}{N-1}}$$

where *N* is the number of sampling points. In addition, the range, *Ra*, of dataset *x_α_* is defined by the following:

(14) $$Ra=\max x_{\alpha }-\min x_{\alpha }$$

## 4. Results and Discussion

### 4.1. Variability Analysis of Moisture Contents of Reference RAP Materials

According to the proportions calculated form the GRA, the four reference RAP materials were produced. For each reference RAP material, moisture content tests were conducted using five 2000 g replicate samples. The moisture content test results and the calculated variability indicators of the four reference materials are presented in Table 7 and Table 8. It can be observed that, compared to the unfractionated Reference-1 material, References-2, 3, and 4 exhibited significant reductions in the *S*, *COV,* and *Ra* of moisture content. In particular, compared to the *COV* value from the commonly used two-fraction processing, those from the four-fraction and six-fraction methods decreased by 39.8% and 47.6%, respectively. This indicates that the refined fractionation processing method can effectively reduce the variability in the moisture contents of RAP materials. As the number of fractions increases, the variability in the moisture content of the reference materials decreases accordingly; however, the extent of reduction diminishes with further increases in the number of fractions.

### 4.2. Variability Analysis of Asphalt Contents of Reference RAP Materials

Asphalt content testing was carried out using five 2000 g replicate samples for each of the four reference RAP materials. Table 9 and Table 10 show the asphalt content measurements and the calculated variability indicators of the four reference materials. It can be observed that, compared to Reference-1, References-2, 3, and 4 displayed a gradual decrease in the *S*, *COV*, and *Ra* of asphalt content. It is worth noting that compared with the *COV* value from the commonly used two-fraction processing, those from the four-fraction and six-fraction methods declined by 39.5% and 51.5%, respectively. This indicates that the refined fractionation processing approach not only effectively mitigates the variability in the asphalt contents of RAP materials, but demonstrates that a greater number of fractions results in improved homogeneity of the RAP material as well, which is consistent with findings from other researchers [4,28,29].

### 4.3. Variability Analysis of Gradations of Reference RAP Materials and Old Aggregates

The old aggregates within RAP play an essential load bearing and transfer role along with virgin aggregates in recycled asphalt mixture. The gradation of the reclaimed aggregates directly impacts the stability of recycled mixture as a structural layer in pavement systems. If the gradation variability of the RAP material is high, it becomes difficult to ensure the consistency of the recycled mixture’s gradation, which is one of the critical factors limiting the percentage of RAP in recycled asphalt mixtures. Therefore, it is crucial to experimentally evaluate the degree of variability in the gradation of RAP materials, as this serves as an important indicator for determining whether the RAP can be successfully utilized in recycled mixtures. In this section, variability assessments are conducted separately on the gradations of the four reference RAP materials and the gradations of the old aggregates within them.

**Figure 3:**
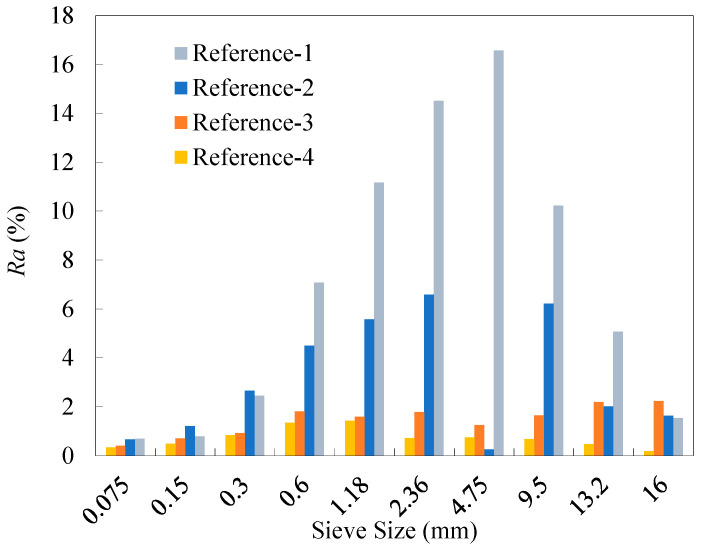
Ranges of the percentage retained of the four reference RAP materials.

Sieve analysis was performed using five 2000 g replicate samples for each of the four reference RAP materials. The specific test results of percentage retained of the RAP particles are presented in Table 11, and Figure 3 gives the range *Ra* (i.e., the difference between the maximum and minimum) of the percentage retained for each of the four reference materials. As observed, the range values of all percentage retained values for References-2, 3, and 4 met the requirement, less than 10%, specified in recycling technical specifications. In contrast, the range values over the sieve sizes of 1.18~9.5 mm for the unfractionated RAP material (Reference-1) exceeded 10%. This suggests that dividing RAP into just two fractions can reduce the dispersion of RAP gradation to a certain extent. In addition, it can be observed from the results for References-3 and 4 that the refined fractionation methods, i.e., the four-fraction and six-fraction approaches, further significantly alleviated the gradation divergence of the RAP particles smaller than the NMAS (13.2 mm). The range values for RAP materials across all particle sizes generally decreased as the number of fractions increased, although the extent of reduction gradually diminished. This implies that a refined fractionation yields lower variability in RAP materials, as well as improved homogeneity of the reference materials.

**Figure 4:**
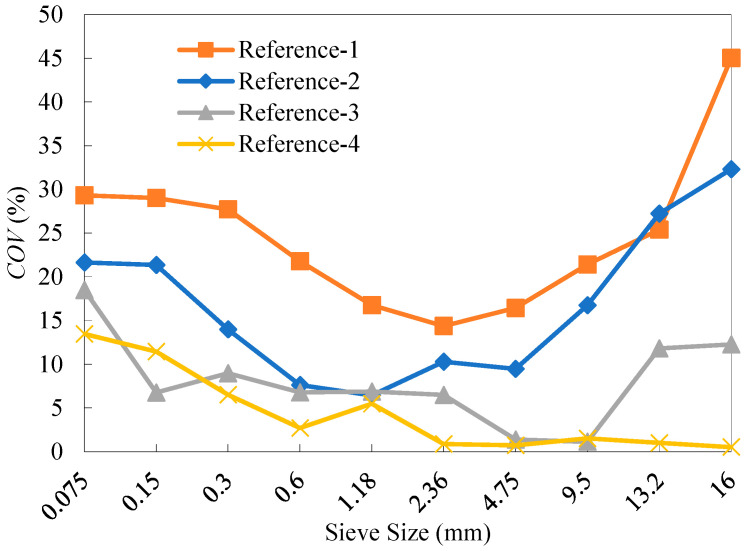
*COV* values of the percentage retained of the four reference RAP materials.

As can be seen from Figure 4, the degree of variability in the RAP materials varies across different particle sizes for the four reference materials. Significant variability in the gradation of RAP materials is primarily observed within the size segments of 9.5~19 mm and 0~0.3 mm, whereas the variability is relatively low in the size segment of 0.3~9.5 mm. In addition, the overall variability of the RAP materials decreased as the number of fractions increased. Compared to the average *COV* value over all the sieves from the two-fraction processing, those from the four-fraction and six-fraction methods decreased by 51.5% and 73.5%, respectively.

**Figure 5:**
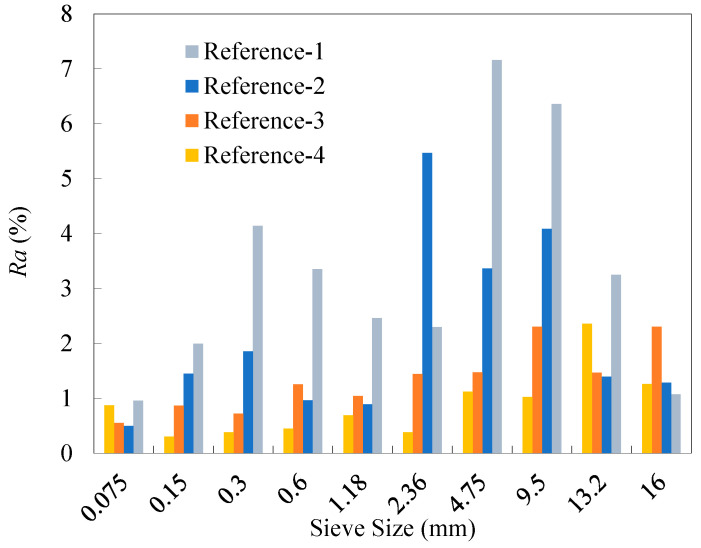
Ranges of the percentage retained of the old aggregates for the four reference RAP materials.

**Figure 6:**
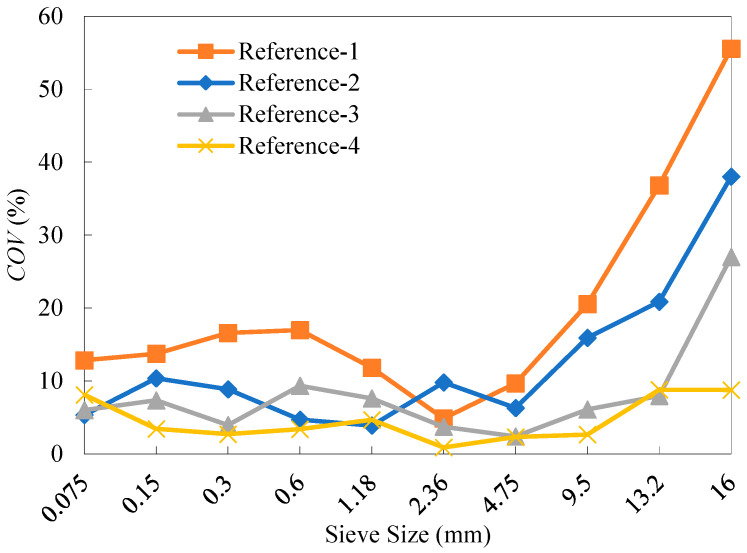
*COV* values of the percentage retained of the old aggregates for the four reference RAP materials.

The old aggregates for each reference RAP material were obtained by the ignition method. The test results of percentage retained from sieve analysis are presented in Table 12. The range values of the percentage retained for the old aggregates from the four reference materials are shown in Figure 5. As can be seen, the range of the old aggregate gradation generally decreased as the number of fractions increased, demonstrating that a higher number of fractions can effectively reduce the gradation variability of the old aggregates. Figure 6 shows the distribution of *COV* values in the gradation of the old aggregates. It can be observed that the fractionation processing effectively reduced the variability of reclaimed aggregates in RAP, with the effectiveness increasing as the number of fractions rose. Compared with the average *COV* value over all the sieves from the two-fraction processing, those from the four-fraction and six-fraction methods decreased by 34.4% and 63.2%, respectively.

**Figure 7:**
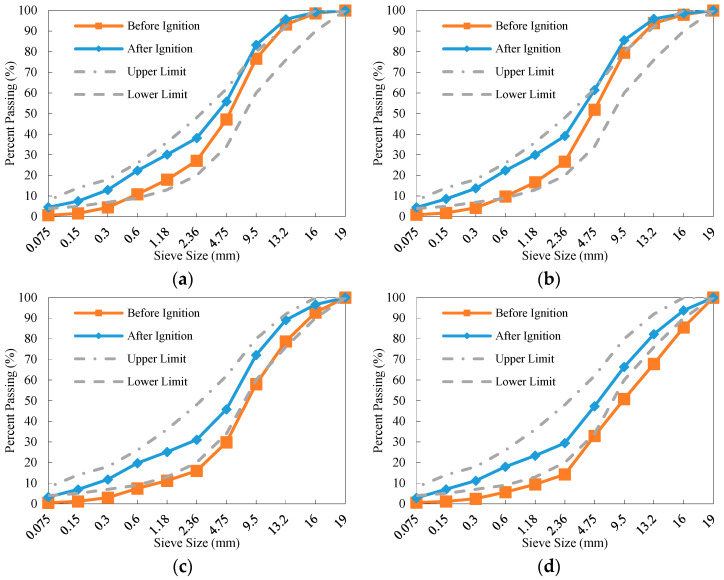
Grading curves of the reference RAP materials before and after ignition: (**a**) Reference-1; (**b**) Reference-2; (**c**) Reference-3; (**d**) Reference-4.

The gradation curves of the four reference mixtures before and after the ignition test are plotted in Figure 7. It can be observed that, compared to the RAP material before ignition, the gradation of the reclaimed aggregates after being burned falls closer within the upper and lower limits specified for AC-16. Furthermore, due to long-term loading and milling operations, some coarse aggregates became fine, though the degree of fragmentation was limited. Consequently, the target gradation can still be achieved by supplementation with additional virgin coarse aggregates.

**Figure 8:**
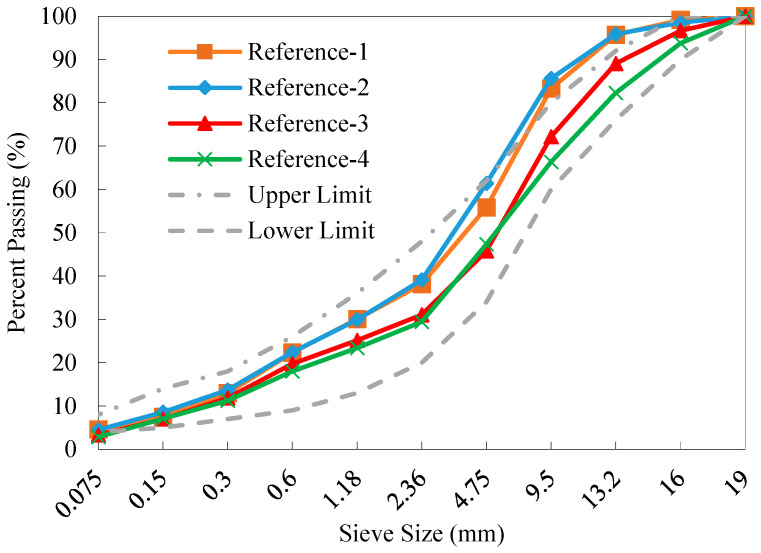
Tested gradations of old aggregates of the four reference RAP materials after ignition.

As shown in Figure 8, the tested gradations of the old aggregates in References-3 and 4 fall within the upper and lower limits specified for AC-16, while the gradation curves of References-1 and 2 for particles above 4.75 mm exceed the upper limit of AC-16. The grading curves of the old aggregates of References-1 and 2 are found very close to each other. This indicates that the widely used two-fraction method cannot effectively eliminate variability in gradation of RAP aggregates. However, as the number of fractions further increased, the gradation of the reclaimed aggregates in the RAP (see References-3 and 4) approached closer to the median of the AC-16 target gradation. This suggests that the GRA method [34] used in this study can effectively blend the fractionated RAP materials based on the particle size distribution characteristics of the reclaimed aggregates. This process significantly controls and mitigates the potential gradation variation issue of RAP materials and enables the high percentage reuse of RAP in recycled mixtures with desirable performance.

This study used RAP material from only one source, but in many cases RAP materials are collected from multiple sources. The findings from this study should be further demonstrated in multi-source scenarios. Investigation into other factors that may impact the variability of RAP properties such as particle agglomeration, asphalt aging, and binder activity is needed in future studies. Furthermore, additional research efforts are required to assess the effect of reduced RAP variability caused by refined fractionation processing on the performance of the corresponding recycled asphalt mixtures.

## 5. Summary and Conclusions

This study investigated approaches for evaluating and controlling variability in properties of RAP materials, including moisture content, asphalt content, and gradation. Four different fractionation methods, i.e., unfractionated, two-faction, four-fraction, and six-fraction processing approaches, were considered, and accordingly four reference RAP materials were prepared by rationally recombining the obtained RAP fractions in different proportions towards the target gradation. The GRA was adopted to identify the correlation between the gradation of old aggregates recovered from each RAP fraction and the target gradation and to determine the optimal proportions of the RAP fractions for different reference RAP materials, thus achieving a satisfactory control on heterogeneity in the gradation of the old aggregates. Various statistical indicators were adopted to assess the variability of RAP properties. Based on the results and analysis, the following conclusions can be drawn:

(1) The unfractionated method and commonly used two-fraction processing struggle to efficiently control the variability of RAP properties, particularly the gradation of old aggregates.
(2) Compared to unfractionated RAP materials, the variability of RAP materials subjected to fractionation processing showed significant improvement. Moreover, as the number of fractions increased, the degree of variability in the RAP materials remarkably decreased. Specifically, compared with the *COV* values from the commonly used two-fraction processing, those from the refined four-fraction and six-fraction processing methods declined by up to 51.5% and 73.5%, respectively.
(3) Fractionating RAP materials into four fractions in a refined manner represented the most economically viable and technically reasonable processing strategy, as it effectively controlled the variability in the properties of the RAP materials.
(4) The GRA affords a simple and effective approach for directly calculating the proportions of RAP fractions in terms of geometric similarity between grading curves without performing a nonlinear minimization algorithm on a target error function.
(5) Proportioning RAP fractions into reference materials with desirable gradations can be a potential approach for controlling the high-RAP recycled mixture with low variability and superior performance.
(6) Additional research efforts are required to investigate other factors that may cause RAP variability, like RAP source, particle agglomeration, asphalt aging, and binder activity. In addition, assessment on the effect of reduced RAP variability caused by refined fractionation processing on the performance of the corresponding recycled asphalt mixtures is required in future studies.

## Figures and Tables

**Table 1:** Properties of the aged asphalt and old coarse aggregates in the RAP material.

Material	Property	Requirement [33]	Measurement
Aged asphalt	Penetration at 25 °C (0.1 mm)	>20	27.9
	Softening point (°C)	—	67.1
	Ductility at 15 °C (mm)	—	89.8
	Apparent viscosity at 135 °C (Pa·s)	—	0.697
Old coarse aggregate	Flat and elongated particle fraction (%)	≤15	14.1
	Aggregate crushing value (%)	≤26	14.4

**Table 2:** Reference RAP materials from different fractionation processing methods.

Reference RAP	Number of Fractions	Particle Sizes for RAP Fractions (mm)
Reference-1	1	0~19
Reference-2	2	0~4.75 and 4.75~19
Reference-3	4	0~4.75, 4.75~9.5, 9.5~13.2 and 13.2~19
Reference-4	6	0~2.36, 2.36~4.75, 4.75~9.5, 9.5~13.2, 13.2~16 and 16~19

**Table 3:** Target gradation and gradations of old aggregates from the six-fraction processing method.

Sieve Size (mm)	Target (%)	Fraction 1 (%)	Fraction 2 (%)	Fraction 3 (%)	Fraction 4 (%)	Fraction 5 (%)	Fraction 6 (%)
19	100.0	100.0	100.0	100.0	100.0	100.0	100.0
16	95.0	100.0	100.0	100.0	100.0	100.0	47.4
13.2	84.0	100.0	100.0	100.0	100.0	43.7	37.7
9.5	70.0	100.0	100.0	100.0	29.2	27.2	30.6
4.75	48.0	100.0	100.0	25.5	16.9	23.3	22.0
2.36	34.0	100.0	22.4	15.3	13.2	20.2	15.9
1.18	24.5	71.2	15.9	13.7	11.3	16.3	11.8
0.6	17.5	46.5	13.5	11.6	9.3	12.8	8.3
0.3	12.5	21.8	9.3	7.7	6.0	7.8	4.4
0.15	9.5	11.4	6.3	4.9	3.7	4.8	2.3
0.075	6.0	7.0	4.8	3.4	2.5	3.3	1.3

**Table 4:** Difference sequences between the target gradation and the gradations of old aggregates from the six-fraction processing method.

Sieve Size (mm)	Fraction 1 (%)	Fraction 2 (%)	Fraction 3 (%)	Fraction 4 (%)	Fraction 5 (%)	Fraction 6 (%)
16	5.0	5.0	5.0	5.0	5.0	47.6
13.2	16.0	16.0	16.0	16.0	40.3	46.3
9.5	30.0	30.0	30.0	40.8	42.8	39.4
4.75	52.0	52.0	22.5	31.1	24.7	26.0
2.36	66.0	11.6	18.7	20.8	13.8	18.1
1.18	46.7	8.6	10.8	13.2	8.2	12.7
0.6	29.0	4.0	5.9	8.2	4.7	9.2
0.3	9.3	3.2	4.8	6.5	4.7	8.1
0.15	1.9	3.2	4.6	5.8	4.7	7.2
0.075	1.0	1.2	2.6	3.5	2.7	4.7

**Table 5:** Maximum and minimum values of the difference sequences of the reference RAP blends.

Material	*d*_max_ (%)	*d*_min_ (%)
Reference-2	52.0	0.3
Reference-3	52.0	0.1
Reference-4	66.0	1.0

**Table 6:** Gray relational coefficients, gray relational degrees, and proportions of all fractions of old aggregates for Reference-4.

Quantity	Sieve Size (mm)	Fraction 1 (%)	Fraction 2 (%)	Fraction 3 (%)	Fraction 4 (%)	Fraction 5 (%)	Fraction 6 (%)
*λ_j_*(1)	16	82.0	82.0	82.0	82.0	82.0	28.1
*λ_j_*(2)	13.2	54.9	54.9	54.9	54.9	31.7	28.7
*λ_j_*(3)	9.5	38.6	38.6	38.6	31.4	30.4	32.2
*λ_j_*(4)	4.75	26.3	26.3	45.9	37.7	43.5	42.2
*λ_j_*(5)	2.36	21.9	63.3	50.7	48.0	58.7	51.6
*λ_j_*(6)	1.18	28.5	70.6	65.0	59.9	71.6	60.9
*λ_j_*(7)	0.6	39.4	85.8	78.9	71.7	83.2	69.0
*λ_j_*(8)	0.3	68.6	89.0	82.9	76.7	83.1	71.9
*λ_j_*(9)	0.15	95.4	89.4	83.5	79.1	83.2	74.7
*λ_j_*(10)	0.075	99.7	99.0	92.1	88.0	91.5	83.2
*λ_j_*	—	55.5	69.9	67.4	62.9	65.9	54.2
*ρ_j_*	—	14.8	18.6	17.9	16.7	17.5	14.4

**Table 7:** Test results of moisture contents of reference RAP materials subjected to different fractionation processing methods.

Reference RAP	Replicate	*m*_w_ (g)	*m*_d_ (g)	*w* (%)
1	1	2000.0	1983.0	0.86
	2	2000.0	1982.1	0.90
	3	2000.0	1981.6	0.93
	4	2000.0	1978.3	1.10
	5	2000.0	1976.5	1.19
2	1	2000.0	1985.5	0.73
	2	2000.9	1987.3	0.68
	3	2001.3	1986.9	0.72
	4	2001.2	1988.3	0.65
	5	2001.2	1989.3	0.60
3	1	2001.7	1996.1	0.28
	2	2000.8	1995.0	0.29
	3	2000.7	1995.6	0.26
	4	2000.9	1995.6	0.27
	5	2000.0	1995.1	0.25
4	1	2001.2	1996.1	0.26
	2	2003.0	1997.6	0.27
	3	2003.2	1998.0	0.26
	4	2004.1	1998.2	0.30
	5	2001.5	1996.1	0.27

**Table 8:** Variation indicators of moisture contents of reference RAP materials.

Material	*S* (%)	$\bar{x}$ (%)	*COV* (%)	*Ra* (%)
Reference-1	0.47	4.47	10.46	1.07
Reference-2	0.16	4.58	3.49	0.44
Reference-3	0.09	4.09	2.10	0.21
Reference-4	0.07	4.00	1.83	0.18

**Table 9:** Test results of asphalt contents of reference RAP materials subjected to different fractionation processing methods.

Reference RAP	Replicate	*m*_1_ (g)	*m*_1_–*m*_2_ (g)	*Pb* (%)
1	1	1905.9	76.4	3.85
	2	1887.2	88.5	4.48
	3	1899.7	74.2	3.76
	4	1879.7	94.3	4.78
	5	1881.6	88.0	4.47
2	1	1887.0	87.5	4.43
	2	1892.5	87.3	4.41
	3	1892.0	85.7	4.33
	4	1899.5	82.7	4.17
	5	1893.0	90.7	4.57
3	1	1914.4	78	3.91
	2	1914.5	79.7	4.00
	3	1909.7	79	3.97
	4	1907.2	78.8	3.97
	5	1919.6	75.8	3.80
4	1	1919.7	75	3.76
	2	1916.3	78.1	3.92
	3	1918.8	76	3.81
	4	1918.1	77.5	3.88
	5	1916.1	77.1	3.87

**Table 10:** Variation indicators of asphalt contents of reference RAP materials.

Material	*S* (%)	$\bar{x}$	*COV* (%)	*Ra* (%)
Reference-1	0.44	4.27	10.31	1.02
Reference-2	0.15	4.38	3.34	0.40
Reference-3	0.08	3.93	2.02	0.20
Reference-4	0.06	3.85	1.62	0.16

**Table 11:** Test results of percentage retained of the reference RAP materials subjected to different fractionation processing methods.

Reference RAP	Replicate	Percentage Retained (%) at Sieve Size (mm)										
		16	13.2	9.5	4.75	2.36	1.18	0.6	0.3	0.15	0.075	<0.075
1	1	1.8	4.5	21.9	35.1	17.0	6.9	5.0	4.2	1.9	0.6	1.1
	2	1.6	5.3	17.3	30.5	20.4	8.8	6.7	5.8	2.5	0.7	0.5
	3	1.8	7.5	17.1	21.8	24.6	10.9	6.5	5.7	2.6	0.9	0.4
	4	0.3	4.0	13.7	28.7	19.0	10.2	9.1	8.9	4.1	1.3	0.7
	5	1.6	5.8	13.0	31.1	19.0	9.4	7.9	7.5	3.1	1.0	0.6
2	1	2.6	2.5	12.7	30.3	24.6	10.3	7.2	5.8	2.4	0.9	0.8
	2	2.0	5.1	14.4	26.6	23.3	10.8	7.2	6.1	2.6	0.9	0.9
	3	2.2	3.9	15.1	27.1	26.4	9.4	6.6	5.1	2.3	0.9	0.9
	4	2.6	3.9	11.5	30.2	29.0	9.2	6.0	4.4	1.8	0.7	0.7
	5	1.0	5.3	17.7	24.1	22.6	10.2	7.1	6.2	3.2	1.2	1.3
3	1	8.1	12.1	20.9	28.7	13.3	5.1	4.1	4.7	1.9	0.6	0.4
	2	5.9	16.5	20.3	28.3	12.6	4.5	3.5	5.0	1.8	0.8	0.8
	3	7.3	13.6	20.9	28.2	14.2	4.8	3.9	4.3	1.8	0.6	0.5
	4	8.0	13.3	20.6	28.1	14.5	5.1	3.7	4.1	1.6	0.5	0.4
	5	7.1	14.6	20.8	27.6	14.7	4.4	3.6	4.1	1.9	0.6	0.6
4	1	14.5	17.5	17.4	17.9	18.7	4.7	3.9	3.2	1.3	0.5	0.5
	2	14.4	17.8	16.9	17.8	18.5	4.4	3.8	3.5	1.4	0.6	0.8
	3	14.6	17.8	17.0	18.1	18.6	5.1	3.7	2.9	1.1	0.4	0.5
	4	14.4	17.5	16.8	18.0	18.9	4.9	3.9	3.2	1.2	0.5	0.6
	5	14.4	17.9	16.8	18.0	18.4	4.8	3.7	3.1	1.5	0.6	0.8

**Table 12:** Test results of percentage retained of old aggregates of the reference RAP materials subjected to different fractionation processing methods.

Reference Material	Replicate	Percentage Retained (%) at Sieve Size (mm)										
		16	13.2	9.5	4.75	2.36	1.18	0.6	0.3	0.15	0.075	<0.075
1	1	1.1	2.6	16.1	31.9	16.3	6.8	6.2	7.4	4.6	2.4	4.6
	2	1.1	3.3	13.2	26.8	18.6	7.7	7.3	8.9	5.3	2.8	4.8
	3	0.4	5.6	12.7	27.0	17.9	7.8	7.1	8.8	5.1	3.3	4.3
	4	0.3	2.3	10.3	24.7	17.7	9.2	9.6	11.5	6.6	2.8	5.0
	5	1.4	3.5	9.8	26.8	18.1	8.7	8.5	10.1	5.8	3.2	4.2
2	1	1.6	2.2	9.1	25.8	21.6	9.2	8.1	8.8	5.0	3.8	4.6
	2	0.8	3.4	11.2	23.3	20.8	9.7	7.6	9.2	5.3	4.2	4.4
	3	1.8	2.8	10.9	23.5	23.2	8.8	7.4	8.1	5.0	4.2	4.2
	4	2.1	2.0	8.2	25.9	25.6	8.9	7.1	7.7	4.4	3.9	4.3
	5	0.9	2.8	12.3	22.5	20.1	9.1	7.7	9.5	5.9	4.3	4.9
3	1	3.2	7.1	17.1	26.7%	14.6	6.0	5.6	8.1	4.8	3.4	3.3
	2	3.8	7.6	17.6	25.7	13.9	5.5	4.9	8.3	5.4	3.8	3.6
	3	2.7	7.1	15.9	27.1	15.0	6.1	6.2	8.0	4.8	3.7	3.4
	4	2.3	7.5	18.1	25.8	15.3	6.4	5.4	7.6	4.6	3.5	3.4
	5	4.6	8.6	15.8	26.6	15.0	5.4	5.0	7.6	4.6	4.0	2.9
4	1	6.9	11.0	16.2	18.8	17.9	6.2	5.2	6.6	4.0	3.8	3.4
	2	5.8	13.0	15.5	18.5	17.9	5.8	5.2	6.9	4.2	4.0	3.3
	3	6.6	10.8	15.6	19.6	18.1	6.4	5.5	6.6	4.1	4.6	2.1
	4	6.2	10.6	16.5	18.9	18.1	6.1	5.7	6.9	4.3	4.1	2.6
	5	5.6	12.1	15.8	19.3	17.7	5.7	5.4	6.8	4.3	4.3	3.0

## Data Availability

The original contributions presented in this study are included in the article. Further inquiries can be directed to the corresponding author.
